# Road to Equity in Maternal and Child Health: Honoring the Past and Blazing New Paths

**DOI:** 10.1007/s10995-023-03761-x

**Published:** 2023-08-14

**Authors:** Diane L. Rowley, Vijaya K. Hogan, Chad Abresch

**Affiliations:** 1 Morehouse School of Medicine, Atlanta, GA USA; 2 Vijaya K Hogan Consulting, LLC, 300 Colonial Center Pkwy Ste 100N, Roswell, GA 30076 USA; 3 CityMatCH, Omaha, NE USA; 4 Department of Health Promotion, College of Public Health, University of Nebraska Medical Center, Omaha, NE USA

**Keywords:** Birth equity, Infant Mortality, Preterm birth, CDC, CityMatCH, Reproductive health, History, Racism

## Abstract

**Purpose:**

This paper is a historical account of an initiative, as recalled by the authors who were directly involved, that brought to the forefront the long-standing and unjust reproductive health inequities in the United States. It is composed of three distinct but interrelated parts that together map the past, present, and future of addressing racial inequities in Maternal and Child Health.

**Description:**

This paper is composed of three distinct but interrelated parts that together map the past, present, and future of addressing racial inequities in Maternal and Child Health. Part I recounts the history and achievements of a Centers for Disease for Control and Prevention initiative in the 1980–90’s, led by the Prematurity Research Group in the Division of Reproductive Health, Pregnancy and Infant Health Branch. This initiative stimulated a paradigm shift in how we understand and address black infant mortality and the inequities in this outcome. Part II illustrates examples of some exemplary programmatic and policy legacies that stemmed either directly or indirectly from the Centers for Disease for Control and Prevention paradigm shift. Part III provides a discussion of how effectively the current practice in Maternal and Child Health applies this paradigm to address inequities and proposes a path for accelerating Title V agencies’ progress toward birth equity.

**Assessment:**

This CDC initiative was transformative in that it raised the visibility of African American researchers, moved the field from a focus on traditional epidemiologic risks such as personal health promotion and medical interventions, to include racism as a risk factor for inequitable birth outcomes. The paradigm examined the specific roles of historical and structural racism, and the racialized, contextualized, and temporal exposures that are unique to Black women’s experiences in the United States.

**Conclusion:**

The initiative radically changed the narratives about the underlying factors contributing to inequities in birth outcomes of Black women, altered the way we currently approach addressing inequities, and holds the keys for transforming practice to a more holistic and systematic approach to building sustained organizational structures in maternal and child health that accelerate the achievement of birth equity.

## Significance

Revisiting a key initiative in Maternal and Child Health history can help to better evaluate and refine current practices so that progress toward the achievement of reproductive justice and equity can be accelerated.

## Introduction

### The problem

Black infant survival in Ohio is 44 years behind what white infants experience today (James, 2023). Further, even as Ohio experiences overall improvements in infant mortality (IM), the Black rate continues to lag behind the White rate and the *pace* of improvement is slower for Black infants than for Whites. Ohio is not alone, as rates in most states mirror this trend. From any public health or human rights metric, this would be seen as unethical and unacceptable, yet it is a pattern that has dominated MCH for more than a generation. Unless a different approach to public health is taken, we know exactly where this road will lead, and 44 years hence, we will still be asking— “what do we do about this gap?”.

It is long past time for a paradigm shift in MCH and in public health that will accelerate progress toward achieving equity. The problems of our past will continue to be problems in the future unless there is a major shift in the way we address inequities. If such a radical paradigm shift seems impossible, we can point to the history of how *several transformative ideas* first became a part of research and practice in MCH (and in public health) and changed the paradigm of how we approach infant and maternal mortality. A body of work conceptualized and carried out by the Division of Reproductive Health (DRH) at the CDC in the 1980–90 s, serves as a historical marker for past transformation, and as inspiration for future transformative change in the field. This historical account serves as a case study developed by three authors, who were directly involved in the work. The first author (DLR) was also leader of the initial activities; the second author (VKH) became the second team leader and carried the legacy to other MCH arenas; and the third author (CA) is the current lead of an organization that translated this research into action in the field.

This report has three interrelated sections. The first section describes the early activities that transpired during the first decade and some of the products connected to the work. These products radically changed the narratives about the underlying factors contributing to inequities in birth outcomes of Black women and altered the thinking and action in MCH. The second section describes programmatic and policy initiatives that developed either directly or indirectly from the CDC paradigm shift, including a focus on how one organization-CityMatCH operationalized this new knowledge. The third section is a discussion of how effectively the current practice in MCH applies this paradigm to address inequities and proposes a framework for accelerating progress toward birth equity in the US.

## Part I: The History of How We Entered: Shifting the Research Paradigm for Black Infant Mortality

Only the BLACK WOMAN can say ‘when and where I enter, in the quiet, undisputed dignity of my womanhood, without violence and without suing or special patronage…’

Anna Julia Cooper.

In 1989, a group of women in the DRH of the CDC, formerly known as the Center for Disease Control until 1992, began the process of broadening approaches to studying underlying contributors to disparities in IM as experienced by Black families. Historical documentation of public health rarely highlights the perspective of the writers. This account does, because the authors want to provide insight into what it was like bringing Black women’s perspective into a federal agency. Our work at CDC was influenced by a cultural perspective that values and centers African American women’s experiences and empowers African American women with the right to interpret their reality and define their objectives (Taylor, 1998). Our intent was to initiate creative actions in a public health research environment within a federal agency where the status quo approach was one of linear thinking about individualistic disease causation under conditions that some African American public health professionals considered to be a hostile environment.

**Fig. 1:**
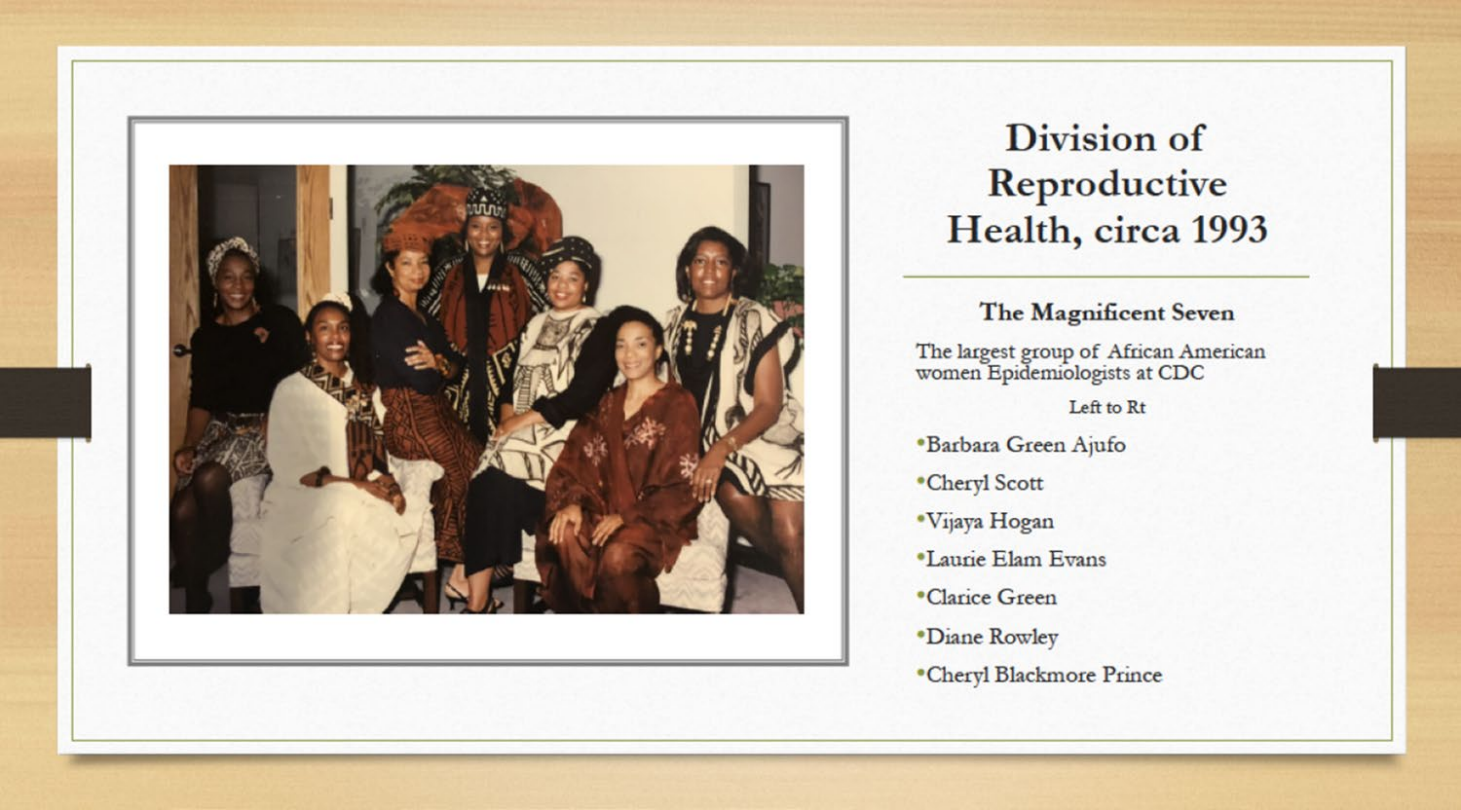
The Magnificent Seven. African American women epidemiologists of the Division of Reproductive Health, National Center for Chronic Disease Prevention and Health Promotion, Centers for Disease Control and Prevention, circa 1993

What follows is a description of the early development of research to eliminate IM disparities, viewed from the perspective of two African American women that had lead roles in research and programmatic processes. Ultimately, the initiative attracted one of the largest cadres of female, African American epidemiologist researchers to work in the DRH and these women specifically focused on perinatal epidemiology (Fig. 1).

### Prologue

In 1988–89, the Task Force on Infant Mortality was convened by President George HW Bush’s Domestic Policy Council. A major impetus for the Task force was the unfavorable international ranking in IM rate of the US. The task force was unique because it functioned with the assumption that all governmental sectors should brainstorm ideas about improving the abysmal international ranking of US IM, and thus, included representatives from nine Cabinet Departments. The task force was headed by James O Mason, Assistant Secretary of Health and Human Services with most of the work being done by three agencies, the CDC, the Health Resources and Services Administration (HRSA), and the National Institutes of Health (NIH). The Task Force work was conducted at the same time the *Healthy People 2000: National Health Promotion and Disease Prevention Objectives* was being developed. For the first time, the reduction of health disparities among Americans was included as one of the three broad goals of Healthy People. As a result, Mason linked the Infant Mortality Task Force report to infant health disparities in a New York Times article saying, “Unacceptable racial disparities and significant geographic inequities are still evident, and progress in reducing the rate of IM has slowed significantly, especially among Blacks” (Pear, 1990).

The Task Force report called for new or expanded funding for activities now considered bedrock MCH structures: annual linkage of infant birth and death certificates, expansion of the Pregnancy Risk Assessment Monitoring System (PRAMS) to a national surveillance program, expansion of the Maternal and Child Health Epidemiology program, and two new activities-the national Healthy Start initiative, and research on the Black/White gap in IM (now referred to as the infant health disparity).

### Black/White Gap Activities, 1990–2001

The first prevention research group that focused specifically on eliminating racial disparities in IM was formed in the CDC DRH, initially under the leadership of James Marks, DRH Director (and later Center Director), Carol J Hogue, Chief of the Pregnancy and Infant Health Branch (and later Division Director), and Hani Atrash, subsequent Branch Chief—all of whom supported the process of developing a new approach to addressing the Black/White gap in IM. Carol Hogue contributed substantially to the strategic thinking of the research agenda. The day-to-day actions were assigned to a planning team that faced the tension of developing a research agenda substantially different from the prevailing public health model in the CDC’s National Center for Chronic Disease Prevention and Health Promotion (NCCDPHP), where DRH is housed. NCCDPHP focused on surveillance of individual behavioral health risk and a prevention strategy that promoted healthy behaviors and clinical preventive services.

The first author (DLR) was also a member of the inaugural board of directors of the National Black Women’s Health Project (NBWHP) and wanted to imbue the NBWHP’s approaches into the research on eliminating health disparities. NBWHP focused on a holistic vision of health that accounted for all aspects of a woman’s life and health (physical, mental, and spiritual) and was concerned about the ways in which oppression experienced due to race, ethnicity, gender, and class intersected in Black women’s lives, affecting their mental, physical, and spiritual health (Hart, 2012). At that time, the emphasis on both integrating physical, mental, and general well-being along with a societal role in determining health status were quite different from the predominant public health model.

Prevailing research and programmatic activity centered on the importance of low birth weight (LBW) as a predictor of IM and of the racial differences in IM (Behrman, 1985; Kleinman & Kessel, 1987). A publication by the Institute of Medicine (now the National Academy of Medicine) on the contribution of LBW to IM called for preventive approaches that ranged from specific medical procedures to broad-scale public health and educational efforts (Birthweight, 1985). The Preventing LBW publication focused on the need to expand the availability of prenatal care service and enhance the content of prenatal care. Most epidemiologic studies of IM among Black women focused on aspects of prenatal care.

Recent findings from a national linkage of 1983 infant birth certificates to infant death certificates had provided new insight about maternal and infant risk factors for IM, LBW and contributors to the higher rate of IM among Black babies (Hogue et al., 1987). One analysis reported that two-thirds of the Black-White gap in IM was due to deaths among very low birth weight (VLBW) Black infants, and since preterm birth (PTB) was associated with most VLBW births there was a crucial need to identify strategies to reduce PTB (Iyasu et al., 1992). However, we were concerned that vital records and other existing epidemiologic data did not capture the range of risk factors that would explain the gap in IM between Black and White infants. Moreover, because the low birthweight included two categories, *small for gestational age* infants and *preterm* infants, one set of prevention strategies was probably inadequate to address both categories.

The first year of the Prematurity Research Group included a study group devoted to brainstorming about the spectrum of contributors to the Black/White gap in IM, the types of studies needed to examine those contributing factors, and the broad range of prevention strategies needed. One member of the assembled team encouraged us to develop separate foci on small for gestational age infants and infants born because of preterm delivery subtypes (Savitz et al., 1991). Other discussions raised the question of whether the contributors to the general population level rates of LBW and PTB were the same factors that were associated with the Black/White gap in mortality.

Another member asserted that there needed to be a fundamental change to the scientific approach to studying health disparities, that we should intentionally embrace Thomas Kuhn’s ideas on the need for a new paradigm because of emerging evidence that challenged the assumption that disparities in LBW and IM rates among African American families were due to lower utilization of prenatal care and lower socioeconomic status (Ferré et al., 2010; Kuhn, 1970). The study by Schoendorf et al. examined the mortality rates and birth-weight distributions of infants whose parents were both college graduates and found the likelihood of death for a Black infant was 1.82 times that for a White infant (95% CI [1.64, 2.01]) (1992).

Results of an analysis of birth weight distribution demonstrated nearly identical mortality rates among infants weighing at least 2500 g at birth, suggesting that the difference between Blacks and Whites was attributable to the much higher incidence of primarily VLBW among Black infants (Schoendorf et al.). The increased risk of VLBW among Black infants in this selected population suggested a basic lack of understanding of the determinants of premature birth and an inability to prevent premature delivery, even in an educated population (Parker et al., 1994).

The prevention research group funded a second study that examined outcomes of the first live births of a population of Black and White college graduates. No previous study had matched reproductive histories of its study population to this degree. Compared with White graduates, Black graduates had 1.67 times the risk of preterm delivery and 2.48 times the risk of LBW (McGrady et al., 1992). Measures of social and economic status differed significantly by race. However, adjustment for these variables did not reduce the estimated risk for Black graduates compared with White graduates. The inability of socioeconomic status, as usually measured, to explain this disparity suggested a need to look at social and environmental mechanisms capable of producing the racial disparity (McGrady et al., 1992).

We realized that research on health disparities needed to expand beyond traditional epidemiologic studies of disease. A central question we decided to explore was: *what is unique about the experiences of Black women that puts them at higher risk for IM?* We used this question as a way of examining the complex interactions of social, environmental, and medical factors among women of color that could contribute to the higher risk of PTB and IM experienced by Black women as compared to White women. We also had to contend with the lack of studies of what the pregnancy experience was like for African American women, from both medical and social contexts. We recognized that these factors included effects of gendered stress and racism. Epidemiologic studies of stress and health outcomes were an established research area; however, at that time racism was not considered an ‘acceptable’ etiologic factor to study. Rather, epidemiologic studies focused on *race* as a risk factor for poor health outcomes (Blackmore et al., 1993). Asking ‘what was unique about Black women…’ gave us and entry into talking about the way these women experience stress and racism and led us to recognize not only the need for ethnographic studies but also the importance of engaging communities in the conduct of our research. We asserted that health disparities research should involve the community in defining and shaping the study agenda and should incorporate the experiential knowledge of women and their communities and should be given meaningful benefits for participating:

- To find out the reasons for the disparity in IM rate, researchers not only have to review the medical history, but also have to chronicle “her story,” the experience of African American women. Scientists cannot create categories of environmental stress a priori and ask women how they fit into the categories and cope with the stressors. The description of the stressors must capture the reality of how women experience their lives.
- To capture this reality, women need to be consulted on the design of the study, on what should be studied, and on how to go about collecting the information. Women should be asked what benefit they would like to get out of the experience.
- Collaborative research with women and their community will help to avoid the dual problems of scientific racism and intellectual colonialism. The Tuskegee Syphilis Study is the most well-known example of scientific racism. When intellectual colonialism occurs, professionals earn their salary, publish, and achieve tenure by using the raw data collected from the African American community, while study participants may receives only a token contribution in return. Even when no direct harm is perpetrated on the study participants, the community receives no direct benefit from the study. (Rowley, 1994)

As a result of expansive readings and discussions with other CDC professionals we broadened our scope to incorporate an understanding of a relatively new field, psychoneuroimmunology. We considered the possibility that the unique phenomena experienced by Black women of chronic stress and racial discrimination could affect the brain, endocrine and immune systems during pregnancy that contribute to a PTB. We decided that a comprehensive understanding of the research in this area could bolster our approaches to advancing strategies to prevent PTBs.

These staff discussions made clear *the need* to reach out for other expertise in sociology, psychology, health policy, obstetrics, and community-based maternal health advocates for additional input. These activities culminated in a decision to stimulate a paradigm shift by convening a conference in 1991, *The Black/White Gap in Infant Mortality.* We commissioned a series of conference papers, intentionally designed to reflect multidisciplinary perspectives, for discussion and feedback. In 1993 many of the papers from the conference were published in a supplement of the *American Journal of Preventive Medicine* (Rowley & Tosteson, 1993).

In addition to our description of a new research strategy that focused specifically on PTB among African American women, the articles in the journal were an intentional shift from the traditional epidemiologically-focused studies produced by CDC to a set of broad ranging discussions that included IM and health policy; a new historical perspective on exclusion of Black infant health from public health; the need to address the conduct of research in that the Black community because of the legacy of the Study of Syphilis in the Negro Male; the importance of community participatory methodology; frameworks and approaches to analyzing social and psychosocial stress in women’s health; and recognition of the need for new research on the combined effects of race, gender and social class on health and well-being (Dressler, 1993; Gamble, 1993; Hargraves & Thomas, 1993; Hatch et al., 1993; Krieger et al., 1993; McLean et al., 1993; Rowley, 1994; Wise, 1993).

From 1990 to 93, a series of requests for proposals and contracts were issued by CDC to conduct (1) qualitative studies of pregnancy among Black women in the US using Community-Based Participatory Research (CBPR) methods; (2) studies on stress among African American women; and (3) research on the physiology of PTB. This work included research on the contexts in which social, behavior, cultural, historical, political, and economic forces influence health during pregnancy and on the incorporation of CBPR approaches. We also worked jointly with the National Institute of Child Health and Human Development to fund research on Sudden Infant Deaths among African American and among American Indian populations (Hauck et al., 2002, 2003; Iyasu et al., 2002).

While NCCDPHP leadership supported these funding opportunities, we were caught off guard by the resistance of the grants management office to receive and conduct reviews of applicants. For example, the pushback on developing a measure of gendered stress and racism came in the form of denial of a receipt of an application, claiming that it arrived a few minutes after the deadline. This resistance delayed the work for a year and was only supported when Carol Hogue joined the research team.

In 1999, a second conference*, The Social Context of Pregnancy Among African American Women: Implications for Preterm Delivery Prevention,* was held to review the state of the science that had evolved, much of it through the CDC funding. That conference endorsed the hypothesis that Black women’s social exposures were directly linked to biological phenomena that cause PTB and that PTB constituted a multidimensional, complex interaction of factors that had to be considered altogether, not piecemeal. These activities solidified the shift from a traditional, epidemiological focus on identifying *risk factors* that predicted health status and health outcomes to the importance of *understanding social environment and social forces* as major determinants of health disparities (Hogan et al., 2001; Hogan et al., 2001; Rowley, 2001).

That conference also produced a set of publications that appeared in the *Maternal and Child Health Journal* in 2001 that began to describe what those social exposures were and to determine how best to measure them using qualitative research and community participation. These publications provided evidence that traditional measures of socioeconomic status and health behaviors during pregnancy did not explain health disparities and that chronic maternal stress, independent of other established risk factors, predisposes women to infection during pregnancy that neuroendocrine, immune/inflammatory, and vascular processes may bridge the experience of social adversity before and during pregnancy with PTB (Berg et al., 2001; Culhane et al., 2001; Wadhwa et al., 2001). They also introduced qualitative methods and participatory research models that involved community members in all phases of the research on the social environment and social forces that are present during pregnancy (Jackson et al., 2001; Mullings et al., 2001; Peacock et al., 2001). More detailed qualitative research and community participatory research findings were published elsewhere from studies conducted in Atlanta, Harlem, New York, and Los Angeles. Both the Atlanta and the Harlem-based research incorporated a critical analysis of the multiplicative impact of race, class, and gender on health into conceptual frameworks for locating causal factors for health disparities.

Research conducted in Atlanta focused on the use of CBPR methods to generate a tool to measure the multiplicative impact of identity stressors connected to race and gender, creating the idea of racialized gender stress (Jackson et al., 2005). The work conducted in Atlanta emphasized the necessity for collaborative research, informed by the lived experiences of women of color and for all collaborators to make immediate use of the findings to design interventions (Jackson, 2002). Measurement of the combination of exposure to racism and gendered oppression as a contributor to health disparities was actualized in a new tool that measured individual exposure to racialized and gendered stress (Jackson et al., 2005).

The Harlem BirthRight project analyzed the ways in which resource inequality, institutionalized racism, and gender discrimination together structure access to such resources as employment, housing, recreation, health care, and supportive relationships, as well as how women confront these constraints (Mullings et al., 2001). The Sojourner Syndrome framework, a survival strategy that described the multiplicative effects of class, race, gender, and history of resistance on health, emerged from this study allowing for the exploration of agency (Mullings, 2000, 2005). The Harlem BirthRight research directed attention away from individual risk factors to the structural constraints and the ways people resist them, both of which have health consequences. It drew attention to the need for large-scale changes that provide access to employment, shelter, education, and health care (Mullings, 2005).

The Los Angeles project resulted in the development of the Healthy African American Families (HAAF) project that pioneered the concept of community-partnered research (CPPR), a model to engage community and academic partners equally in an initiative to benefit the community while contributing to science (Ferré et al., 2010; Jones et al., 2009). This form of CBPR became a community-oriented, self-help mechanism for directing power, collective action, system change, social justice, and civil rights in addressing health disparities at the local level. While HAAF originally focused on pregnancy experiences, reproductive health was never viewed as separate from other health or community issues. Reproductive health became integrated with other health aspects, in psychosocial and environmental contexts, within the family and the community, and across the life course. Ethically, HAAF presumed that all these issues needed to be addressed (Ferré et al., 2010).

This body of work brought a spotlight to the contextual differences in history, exposures, power, and resources experienced by Black women, and linked the differential exposures to inequitable outcomes. It redirected efforts to address health disparities beyond access to prenatal care strategies. It was important to shift the focus away from individual care toward a broader understanding of the complex array of social, structural, and historical factors that weave the web of inequities in birth outcomes. For the first time, the public health model for maternal and infant health shifted from planning single factor interventions to consideration of how to address the social processes that influence health outcomes.

### From Health Disparities to Health Inequities

During the 1990–1999 decade, our effort to shift the paradigm on preventive research on infant and pregnancy health disparities had several impacts:

- It was successful in creating a new focus on examining the etiology of PTB to explain the Black/White gap in IM.
- In retrospect, it was an early example of the need to study the intersection of race, gender, class.
- It expanded the importance of understanding the contribution of social factors and social context when addressing the elimination of population disparities.
- It encouraged and supported research that extended beyond studying individual acts of racial discrimination to the societal impact of racism.
- It expanded the importance of understanding and addressing historical contributions to current inequities,
- It highlighted the necessity of not only community partnered research, but also of community-initiated, community-driven research and lastly,
- It emphasized the importance of conducting qualitative studies as part of the framework for understanding pregnancy health outcomes.
- This historical account demonstrates the multiplicative benefits of engaging scientists of color, supporting their creativity, enabling them with resources and positional leadership, and facilitating thought partnerships across agencies, sectors and with communities to ensure dissemination and interpretation of research.

This work also portended the shift from a focus on health disparities to the focus on health inequity as defined by the World Health Organization (WHO), Commission on the Social Determinants of Health (CSDH). During the time we were developing our research agenda, the current concept of health equity emerged from the writings of Margaret Whitehead who noted that health inequities were avoidable and preventable, and therefore were unjust (Whitehead, 1991). Whitehead’s work was recognized in 2005 when the WHO launched a focus on health equity with the establishment of the CSDH to address the social factors leading to ill health and health inequities, and to draw the attention of government agencies and policy makers to the social determinants of health (SDOH) (Fee & Gonzalez, 2017; Solar & Irwin, 2010). In the US, Braveman linked the need to eliminate health disparities with the concept of health equity (2014).

The groundbreaking work of the Prematurity Research Group in the DRH, Pregnancy and Infant Health Branch radically changed the narratives about the underlying factors causing inequities, and about the necessary approaches to achieve equitable birth outcomes. Additionally, the work led to the development of the initial perceived racism impact scales, which have become widely used in subsequent MCH equity research and community assessments nationally. Consequently, we assert that this initiative was a spark that fueled many subsequent efforts that led to a better understanding of how to systematically address the contributors to health inequities using a more holistic approach.

## Part II: From Paradigm Shifts to the Field: Impact of this Work on Current Understanding and Approaches to Addressing Inequities in Maternal and Child Health

As many of the researchers in the field associated with the CDC’s Prematurity Research Group described above are near retirement, have retired, or passed away, it is an appropriate time to recount the history and mark the *legacy* of their work. Moreover, it is also important to examine how this work has proceeded and influenced the field and how it has been translated into practice. There are several areas where this work has made significant impact. It has constituted other lines of thought (i.e., furthering the paradigm shift) to produce an enhanced understanding of the true complexity of the challenge of health inequities (e.g., life course, social determinants, health equity, intersectionality, critical race theory). It has launched transformative practice within public health agencies to eliminate inequities in MCH (e.g., CityMatCH, the Association of Maternal and Child Health Programs, the Association of Teachers of Maternal and Child Health, UNC-MCH-Workforce Development Center, Michigan Public Health Institute, Achieving Birth Equity through Systems Transformation, Michigan Department of Health and Human Services Practices to Reduce Infant Mortality through Equity *(*PRIME), shaped the conceptualization and implementation of new initiatives such as Healthy Start, increased the number of Black female researchers, and raised the profile of several incredible researchers (e.g., Camara Jones, Fleda Mask Jackson, Dara Mendez). Lastly, it has nudged funders, academic systems, practitioners, and political leaders to take notice of the ways their systems and processes have continuously thrown barriers in the path to equity, encouraging these organizations to support innovative processes and approaches (e.g., rise in anti-racism trainings, Nurture New Jersey) (Nurture NJ, 2021). In this way, the bold work of the Black women in CDC’s Prematurity Research Group served as the bedrock that transformed the field of MCH from one stuck in medical and behavior health models to one emphasizing socio-structural drivers of health inequities.

You may shoot me with your words,

You may cut me with your eyes,

You may kill me with your hatefulness,

But still, like air, I’ll rise.

….. Maya Angelou

Given the transformative role the work of the Prematurity Research Group played in the field, it is important to examine some of its legacies. These are summarized in Table 1 and cover both direct and indirect legacies of the work. Taken together, these encompass a great deal of systemic change within the field of MCH, including an enhanced focus on community partnerships to address health inequities, the incorporation of qualitative research and epidemiological methods, and goal shifts away from reducing health disparities and toward eliminating health inequities.

**Table 1:** Summary of key legacies related to the work of the prematurity research group, division of reproductive health, national center for chronic disease prevention and health promotion, centers for disease control and prevention

Legacy	Description
Direct legacy	
Community Partnership in Public Health Planning	The inseparability of individual health from the community and environment in which individuals live, work, and play, and hence the importance of community partnerships emerged as a theme during the 1997–99 development of the Healthy People 2010. There has been a significant uptick in the number of requests for proposals that include a requirement for community engagement since the 1990’s
Redirection of Focus on Elimination vs Reduction of Health disparities	Two goals were identified for Healthy People 2010, one of which was not just a reduction, but the elimination of health disparities. The elimination of health disparities required a more expansive view of the contributing factors and a more structural approach to achieve a permanent elimination of disparities. The CDC work laid some groundwork for approaches to eliminate disparities. Although not clearly defined, achieving health equity was included under the goal of eliminating health disparities. (https://www.paho.org/hq/dmdocuments/2010/National_Health_Policies-United_States-Healthy_People_2010.pdf, Assessed 4/3/2021)
Expansion from Individual Risk to Social Determinants and Place-based work	The CDC initiative both directly funded and inspired several studies on the importance of a shift in thinking about discrete interventions to creating the social circumstances that support healthy birth outcomes. (Place-based approaches, research on neighborhood factors and health outcomes, research on racism and health, etc.)
Qualitative Research in MCH	There has been a significant uptick in the number of studies that include qualitative research or mixed methods since the 1990’s. The CDC initiative midwifed the birth of qualitative research as a valid approach in MCH
Alignment with Other Emerging Theories to Improve Understanding of the Complexity of Health Inequities	The CDC initiative opened a door for the emergence of new ways of thinking about inequities and approaches to address them (e.g. Critical Race Theory (CRT) and health inequity (Ford & Airhihenbuwa, 2010); Lifecourse theory (Lu & Halfon, 2003) Intersectionality policy toolkit (Bowleg, Hull et al., 2020)
State Plan Impacts	One example of this impact can be seen in the Nurture-NJ Statewide Strategic Plan. In 2020, New Jersey ranked 47th highest in maternal deaths and has one of the largest disparities between maternal outcomes for Black compared to other birthing people. The state’s First Lady initiated Nurture NJ to reverse this trend and to make NJ the safest place in the US to give birth. The Nurture NJ program encompassed the collection of efforts that included a comprehensive strategic plan that encompassed community power building; an antiracism focus; health, social and organizational transformation; as well as several transformative health and social policy initiatives in support of maternal health signed by the Governor. (https://nurturenj.nj.gov)
Indirect legacy	
State, Local and Organizational racism declarations	As of August 2021, 209 declarations of racism as a public health crisis have passed in 37 states. These declarations were adopted by city/town councils, county boards, governor/mayoral statements, education boards (e.g., school boards), and health associations or public health departments. (https://www.apha.org/topics-and-issues/health-equity/racism-and-health/racism-declarations)
Recognition of inequities in funding to Black Researchers	In the 2011, Ginther et al. reported a significant racial gap apparent in NIH R01 funding. That report noted the funding rate for R01 applications from Black/African American scientists was 10 percentage points lower than for all other groups after controlling for an applicant’s educational background, country of origin, training, previous research awards, publication record, and institution characteristics. This wake-up call urged NIH and the biomedical community to look closely at individual and systemwide potential contributors and solutions, codified in 13 recommendations by the NIH advisory committee to the director (Racial Disparities in NIH Funding|SWD at NIH)
Presidential Executive Order on Racism	In January 2021, U.S. President Joseph Biden issued Executive Order #13,985 stating “It is therefore the policy of my Administration that the Federal Government should pursue a comprehensive approach to advancing equity for all, including people of color and others who have been historically underserved, marginalized, and adversely affected by persistent poverty and inequality. Affirmatively advancing equity, civil rights, racial justice, and equal opportunity is the responsibility of the whole of our Government.” We believe the CDC initiative paved the way toward making racism a part of national discourse

The impact of the CDC Prematurity Research Group can be seen not only in its direct work and accomplishments, but in the notable influence it has had on the field of MCH. This is evidenced in organizational- and systems-level change efforts to better support and sustain equity in IM and maternal mortality (MM). While Table 1 lists some indirect impacts of the CDC paradigm shift, a direct example of how the group’s work has influenced the field is exemplified by CityMatCH. CityMatCH is a membership organization of MCH programs within urban health departments. Three CityMatCH initiatives, each bearing the marks of the Prematurity Research Group’s influence, are described below.

### CityMatCH Initiatives

#### BEST Cities

Beginning in 2013, CityMatCH worked to incorporate advances in equity thinking and practice into its work with local health departments and their community partners. Eventually three projects would be rolled up into the Advancing Birth Equity Strategies Together (BEST) Cities initiative. The first project–The Institute for Equity in Birth Outcomes–asked local health departments to partner with community members and organizational stakeholders to plan and implement “downstream” and “upstream” programming to improve equity in birth outcomes. Downstream efforts were often clinical intervention designed to produce measurable impact over the course of 18–24 months, whereas upstream efforts aimed at the root causes of inequities, including entrenched systematic racism. Evaluation data demonstrated a hunger among public health professionals and community members to jointly address birth equity, with over 30 new equity programs implemented in seven participating communities over a two-year period (Collie-Akers, et al., 2021).

#### Racial Healing Revival

A second program from CityMatCH tasked local health departments with collecting oral histories of neighborhoods in their jurisdictions impacted by birth inequities. The oral histories sought to uncover the legacy of municipal assaults, grounded in structural racism, that had been perpetrated against neighborhood residents. Participating health departments unearthed events such as highway projects that bisected neighborhoods and desolated community cohesion, or street closures that effectively sealed off neighborhoods, intentionally isolating residents and reducing their access to the available resources of the larger metropolitan area. Elder residents who lived through these events shared their stories, *as* first-hand accounts*,* that laid bare multigenerational policy efforts that produced present-day birth inequities. The goal of this work was to provide direct education to the health departments from community members.

#### Best Babies Zone (BBZ)

Best Babies Zone was built on Life Course Theory by implementing a place-based strategy to reduce inequities in birth outcomes (Pies & Kotelchuck, 2014). Key to the work was the selection and involvement of a neighborhood with inequitable outcomes. Residents selected the strategies that would be implemented, which often focused on improvements to the built environment, enhanced educational opportunities, or even door-to-door outreach to build a national backbone organization focused on SDOH. The backbone organization would bring participating zones together and provide program structure and training. Ultimately, BBZ proved effective in innovating on traditional public health strategies in ways that were directly aligned with the new emphasis initiated by CDC’s Prematurity Research Group. Participating zones experimented with approaches that were novel to public health practice such as human-centered design, storytelling, and growing public health social movements (Pies et al., 2016). BBZ stands as an early example of MCH infant and MM efforts based primarily on addressing non-clinical, upstream SDOH factors.

### Understanding Impacts

Given the influence the CDC Prematurity Research Group’s efforts had on the field, questions about its “measurable” outcomes are common. Indeed, critics have noted the early primacy and influence of the Prematurity Research Group but have still demanded evidence of its impact on population-level indicators of MM, IM, and PTB inequities in the US. It must be noted that such indicators have not significantly improved over the past three decades (Hill et al., 2022).

In the authors work across our country over many years, we have far too frequently encountered critics of our work and the paradigm shift it produced. These critics fall into two categories—public health practitioners who tacitly acknowledge the need to address SDOH, and influential, generally privileged, community representatives who lack this basic understanding of the core drivers of population-level health outcomes. Invariably, our public health critics ask,: “If a body of work cannot be linked to reductions in inequities in MM, IM, or PTB, how can it be deemed impactful?” Our response is simple, “As a field, our ways of measuring impact require improved sophistication.” Here again, we seek to flip the paradigm by questioning the critics: “Why would you expect that a limited number of non-clinical SDOH interventions, which have been poorly funded and inconsistently applied, would be sufficient to overcome generations of past and current structural racism?”.

On the other hand, critiques from influential, privileged community representatives are more straightforward. As one prototypical critic objected following a keynote presentation, “What you talked about today may be true in other states, but here everyone has the opportunity to be healthy, we just have certain populations who don’t lead healthy lifestyles or comply with care.” Regardless of the whether the critiques are coming from public health professionals or from prominent community representatives, in each case their intended prescription is to move away from population-level impact and toward clinical intervention and counseling/education aimed at individual behavior change (Frieden, 2010). However, at this stage in our field’s progression, the paradigm shift ushered in by the Prematurity Research Group is well established and coordinated efforts could be in place to evaluate and enhance its impacts on ushering in advancements in health equity.

The impact of the Prematurity Research Group’s work on birth equity evokes an analogous image–that of the COVID-19 pandemic-era ship, the *Ever Given*. (Yee & Glanz, 2021). During the pandemic, the *Ever Given*—a member of the class of ultra-large container vessels (ULCVs)—got stuck in the narrow waterway in the Suez Canal. For six intense days, rescue crews dug, drilled, and pulled while the world watched the spectacle with intense fascination. Every day the *Ever Given* blocked the canal, dozens of ships carrying billions of dollars’ worth of cargo were stuck. Once the initial blockage occurred, supply chain delays reverberated throughout the world for months after the incident. The success of finally moving the ship stopped further damage, but the downstream effects did not immediately disappear.

The story of the *Ever Given’s* entrapment is analogous to the stagnation in birth equity we have been experiencing for generations. The walls of the canal can be seen as symbolizing the entrenchments of longstanding societal racism, creating and perpetuating health inequities. Successfully moving the *Ever Given* and unblocking the shipping routes is analogous to the paradigm shift initiated by the Prematurity Research group—progress is evident, but the downstream effects have yet to unfold. Ultimately, something more is needed to clear and remediate the effects of the accumulating downstream blockages. While the portfolio of the research designed, funded, inspired, and supported by the Prematurity Research Group at CDC may have yet to directly result in sustained population-level reductions in MM, IM and PTB inequities, the paradigm shift it created can be seen through its legacies at least as a force that has begun to unblock several major channels leading to health equity. It becomes our task now to continue the legacy with intention to completely unblock the barriers to birth equity.

## Part III: Looking Ahead: Accelerating Momentum Toward Birth Equity

### The Equity Challenge of the Next Decade

The road from transformational research, to practice, to changed population outcomes is a long and arduous one, particularly in the case of eliminating health inequities. The bottom line is we have not made enough of an evolutionary leap from research to practice, and therefore, equitable outcomes will continue to be elusive until we do. There are concentric circles of influence (see Bronfenbrenner, 1977) affecting the actions of public health workers such that the decision-making *behaviors* of public health practitioners, planners, and policymakers are nested within mindset limitations, contextual challenges, and policy and resource limitations. Improvements in birth equity are likely to remain stagnant until multiple levels of individual, structural and positive contextual changes occur to support the ability of public health organizations to make decisions that promote a *culture of equity*. Herein lies the challenge of the next decade: *making a decisive leap from the transformational knowledge we have access to and translating it into a consistent institutional equity infrastructure and practice in MCH.*

### The Complexity of the Challenge

As revealed by the CDC paradigm, health inequities are outcomes that are an amalgam of exposures from the past and the present. Achieving health equity depends on addressing these past and present exposures, as well as ensuring a future that does not reproduce risk exposures for a new cycle or a new generation (Hogan et al., 2018). We now acknowledge that engaging with people with lived experience in communities is paramount for identifying the past and present exposures, conditions, choices, and experiences that constrain health and healthy choices—sometimes beneath the visible part of the iceberg-for the most vulnerable populations. This engagement is also critical for identifying (non-de-) stabilizing *solutions* that address the challenges communities face.

We also know that social determinants of health (SDOH) must be addressed, not just at the individual level where they achieve a temporary reprieve for one person, but at the structural level to ensure we stop the reproduction of risk exposures for entire populations. Further, we know that one agency or organization cannot do all of this restructuring alone and that multi-sector partnerships are necessary to achieve the collective impacts required to make a substantive difference in the contexts of communities. But, since we know all of this, what has gone awry in our ability to achieve birth equity?

### Shortcomings of Current Practice for Addressing Inequities in MCH

The fact remains that the pace of progress toward eliminating disparities in birth outcomes has been unethically slow, unstable, and thus continues to be derailed by shifting political winds. While well-intentioned, MCH has not yet developed and adopted an effective theory of change to achieve equity, nor has it been able to deepen or sustain whatever efforts it does implement. There are several possible specific contributors to the slow crawl toward birth equity in the US, such as:

1. Public health workers may be ill-equipped and often powerless to alter social conditions *on their own*. We need an approach to eliminating inequities that is firmly rooted in the areas of control and influence of MCH (direct service), paired with an approach that does not expend inefficient energy or apply a colonizing mindset to the wider areas of “concern” (e.g. SDOH),—where we have little power of direct influence within the current institutional confines in which we operate (see Covey, 1991). Attempts to address SDOH and other upstream factors can result in more harm when conducted through a non-transformative mindset or a non-equitable lens. The areas where Title V and other MCH workers can have direct transformative impact is in taking a critical approach to their own mindsets, decisions and actions. These mindset transformations should impact the processes and policies under which data are collected and decisions are made that affect population equity. The more public health workers advocate for and adopt equity promoting mindsets and practices (equity in all actions), the more likely they are to move their organizations to make the structural changes required to institutionalize a culture of equity. So, centering equity first and foremost requires turning the lens inward and transforming how each of us as individuals and as organizations make decisions to ensure that they always promote and never inhibit equity.
2. Public health has become politicized to an extent that appropriate action is stifled (Shaw, 2021). The hysteria in many states about “critical race theory” results in often reactionary moves to undermine any discussion of contextualized or racialized experience, which are necessary to understand and address inequities in health. This creates a climate where public health workers feel incapacitated to address the very real underlying cause of a major public health problem and may face personal or professional economic repercussions if they continue this line of work. Programs can be defunded or underfunded, champions can be fired or re-deployed, but establishing an institutional and ultimately a community culture of equity will provide more resilience to shifting political winds.
3. Whether intentional or not, many efforts to address inequities end up being merely performative—they look like the right things to “do” but have little impact because they are not conceived within a holistic ecosystem where one action can inadvertently create collateral damage to another, and we have not “built” any structures to support the ability to continue centering equity. A “Doing” approach to equity usually involves de-contextualized and often siloed actions that may stem from a checklist of necessary “equity actions”, but which may not be sufficient by themselves to move the needle toward equity. For example, increasing the number of grant applications submitted from historically underrepresented groups *without* assembling reviewers who understand the complexity and the context of the work—will not likely increase the number of funded projects from historically underrepresented groups, and in the end will result only in performative action with little or no positive impact, and may possibly cause harm to the populations who are supposed to be helped.

In sum, current equity work appears to be imprisoned by the very structures that need to be transformed: “I know it needs to be done; but I can’t do it because… *(insert structural barrier here)*.” As a field, we need to step out of our structural prisons to be able to apply the new paradigm and employ it through sustained action. This clearly requires work to transform our own institutions to ensure they are adequately supported and empowered to promote equitable information gathering, processes, policies, and decision-making. Innovative leadership and a willingness to maintain a focus on the science, as it develops, is a condition for being able to step out of these structural prisons to create change. This type of internal organizational transformation is a prerequisite to working with communities and other external partners effectively to address the broader social conditions in which inequities are nested. The aforementioned CDC initiative was a model for this type of innovative leadership in that the team “walked the talk,”: was aggressive at self-examination and transformation of their own existing biases, was fearless in defending against opposition to proposed transformations with science, had clear frameworks to counter the headwinds they experienced from critics previously described, and actively pushed to achieve structural changes within CDC and partner organizations to institutionalize a culture that supported and promoted equity.

### A Different Way Forward

We propose that, in contrast to a “doing” equity approach, a *“building” approach to equity* would create a more strategic, creative, holistic, and sustained path. Instead of chasing individual inequities or going all-in on single strategies, individuals and organizations should instead focus efforts on building their institutional ecosystem to support and promote equity (a culture of equity) as a primary outcome objective. A culture of equity exists when the structures, processes, decisions, and behaviors of any given organization are not “equity-neutral” and are intentionally designed to *always* promote and never inhibit equity. We posit that an ecosystem approach is the next logical application of the paradigm shift and is the path to accelerating progress toward birth equity.

#### Equity ecosystem approach

An ecosystem approach promotes the integrated building, coordination and management of multiple and complex pathways and conditions affecting a population to ensure equity and thriving for all. In public health, an ecosystem map provides an important blueprint that shows what conditions need to be *built* in an organization or a community to support equitable birth outcomes. An ecosystem map differs from a logic model, a strategic plan or other planning tool in that it is more holistic and should precede and guide the development of each of these. An ecosystem map is particularly critical when the health conditions and the outcome are very complex, and when it will take a long time before results materialize. In these cases, it is important for multiple partners, within and across many sectors, to keep focused, constant, and consistent in building the necessary conditions for health. The ecosystem becomes the vision for a future that needs to be built to achieve health equity. Since no one stakeholder in a community is likely to be able to build all aspects of the ecosystem, the Ecosystem map can serve as a playbook that choreographs the actions of individual organizations or multiple stakeholders who are working within and across their field to build this health-supporting ecosystem. There are several emerging efforts in MCH using an ecosystem approach that bear watching:

1. *Nurture New Jersey*. This initiative is a statewide birth equity strategic plan that is based on developing an ecosystem across the state that promotes birth equity. (https://nurturenj.nj.gov/).
2. Michigan Public Health Institute’s *Building Community Architecture to Support and Sustain Equity Across Sectors* project, funded by Michigan Health Endowment Fund, is using an ecosystem approach to build institutional and community processes across sectors so that actions and decisions impacting the target communities will be choreographed to collectively build the conditions for equity. A guide for developing a community driven ecosystem map is being developed and tested in two local communities.
3. AMCHP and University of N. Carolina at Chapel Hill’s Systems Mapping to Promote Birth Equity (funded by Pritzker Family Foundation). This project developed a birth equity ecosystem map and will overlay an existing systems map to highlight how MCH funders at the private, community, state, and federal level can better deploy and maximize resources through key leverage points to build an ecosystem that will address all of the health and social challenges impacting infant and maternal health equity.

These examples all take the paradigm to a new level and need to be studied, emulated and improved upon. MCH organizations will accelerate progress toward birth equity when we are able to root out inequities from within our own operating systems and when we create the organizational and collaborative conditions where all of our actions and decisions are centered in equity.

## Conclusion

This article has examined the past, present, and future conception of MCH health equity. By embracing this three-fold structure, we sought, in Part 1, to convey the historic importance of the Prematurity Research Group and the works it spawned. This work has moved the proverbial equity needle to a place where achieving equitable outcomes became possible. In Part II, we explored the present state of MCH equity practice, and the transformations associated with the Prematurity Research Group’s work. The work of CityMatCH, as one example of this translation into MCH practice, created state-based equity implementation zones that could be observed to determine how to improve the application of the paradigm to accelerate progress toward equitable outcomes. Finally, in Part III, observation of the work and impacts of these and other efforts provided insights that help to refine the path to accelerated progress. We define a path that starts with developing transformational mindsets and then by building MCH organizational structures that provide a sustained culture of equity; then collectively impacting the broader social structure as the culture of equity snowballs across MCH organizations and its partners.

While the Prematurity Research Group guides the transformation of current practice to a more holistic and systematic approach to building Maternal and Child Health organizational structures that support birth equity, it is up to each of us in the field to now make the leap into transformative action and structural change by revisiting the example and lessons learned from the history of the Prematurity Research Group and by focusing on the areas where we have the most influence and control, that is building our own individual and institutional structures and capacities to ensure that equity is centered in all policies and practices.

## Data Availability

Not applicable.

## Code Availability

All research cited herein was conducted in accord with prevailing ethical principles and reviewed by an Institutional Review Board.
