# Correction to: Road to Equity in Maternal and Child Health: Honoring the Past and Blazing New Paths

**DOI:** 10.1007/s10995-023-03769-3

**Published:** 2023-09-07

**Authors:** Diane L. Rowley, Vijaya K. Hogan, Chad Abresch

**Affiliations:** 1 Morehouse School of Medicine, Atlanta, GA USA; 2 Vijaya K Hogan Consulting, LLC, 300 Colonial Center Pkwy Ste 100N, Roswell, GA 30076 USA; 3 CityMatCH, Omaha, NE USA; 4 Department of Health Promotion, College of Public Health, University of Nebraska Medical Center, Omaha, NE USA

**Correction to: Maternal and Child Health Journal**

10.1007/s10995-023-03761-x

The original version of this article unfortunately contains error in Funder name in the text. 

In “Equity ecosystem approach” subsection, the funder name “Pritzker Family Foundation” should be changed to “Michigan Health Endowment Fund”, so the complete sentence, reads as follows:

Michigan Public Health Institute’s Building Community Architecture to Support and Sustain Equity Across Sectors project, funded by Michigan Health Endowment Fund, is using an ecosystem approach to build ….

The original article has been corrected.

